# The h-Current in Periglomerular Dopaminergic Neurons of the Mouse Olfactory Bulb

**DOI:** 10.1371/journal.pone.0056571

**Published:** 2013-02-13

**Authors:** Angela Pignatelli, Mirta Borin, Alex Fogli Iseppe, Cristina Gambardella, Ottorino Belluzzi

**Affiliations:** Dipartimento di Scienze della Vita e Biotecnologie, University of Ferrara and Istituto Nazionale di Neuroscienze, Ferrara, Italy; University of Milan, Italy

## Abstract

The properties of the hyperpolarization-activated cation current (I_h_) were investigated in rat periglomerular dopaminergic neurons using patch-clamp recordings in thin slices. A reliable identification of single dopaminergic neurons was made possible by use of a transgenic line of mice expressing eGFP under the tyrosine hydroxylase promoter. At 37 °C and minimizing the disturbance of the intracellular milieu with perforated patches, this current shows a midpoint of activation around −82.7 mV, with a significant level of opening already at rest, thereby giving a substantial contribution to the resting potential, and ultimately playing a relevant function in the control of the cell excitability. The blockage of I_h_ has a profound influence on the spontaneous firing of these neurons, which result as strongly depressed. However the effect is not due to a direct role of the current in the pacemaker process, but to the I_h_ influence on the resting membrane potential. I_h_ kinetics is sensitive to the intracellular levels of cAMP, whose increase promotes a shift of the activation curve towards more positive potentials. The direct application of DA and 5-HT neurotransmitters, physiologically released onto bulbar dopaminergic neurons and known to act on metabotropic receptors coupled to the cAMP pathway, do not modifythe I_h_ amplitude. On the contrary, noradrenaline almost halves the I_h_ amplitude. Our data indicate that the HCN channels do not participate directly to the pacemaker activity of periglomerular dopaminergic neurons, but influence their resting membrane potential by controlling the excitability profile of these cells, and possibly affecting the processing of sensory information taking place at the entry of the bulbar circuitry.

## Introduction

In the olfactory bulb (OB) dopaminergic (DA) neurons represent a fraction of the cells located in the most external (glomerular) layer [1]. In this region populated by three types of interneurons, i.e. periglomerular (PG) cells, short-axon cells and external tufted (ET) cells- often collectively referred to as juxtaglomerular cells - an estimated 10–16% of the neurons in adulthood are positive for tyrosine hydroxylase (TH) [2]–[4], the rate-limiting enzyme for dopamine synthesis. Dopaminergic neurons in the glomerular layer, which include PG cells [5], [6] and a fraction of ET cells [1], have been the object of several studies focused on their role in olfactory signal processing. In spite of the many experiments carried out by a number of different approaches including immunohistochemical [7], [8], behavioral [9], and electrophysiological techniques [10]–[12], their role is far from being understood.

**Figure 1:**
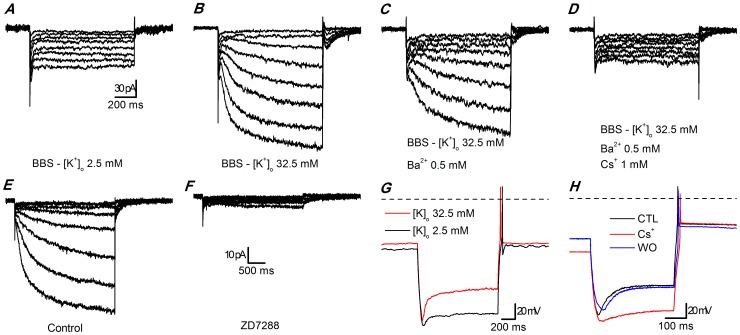
Hyperpolarization-activated currents in slices. A-D: Currents activated by hyperpolarizing steps. A - standard EC saline (EC 1, with TTX 0.6 µM, Cd^2+^ 100 µM); B - high K^+^ EC saline (EC 3, with TTX 0.6 µM, Cd^2+^ 100 µM); C - same as B plus 0.5 mM Ba^2+^ to block the KIR current; D - same as C after addition of a blocker of the h-current (1 mM Cs^+^); the recordings were taken after 5 min at any change of the bathing conditions. All the recordings of this group were performed with BL1 mix; perforated patch in slice at 34 °C.E-F: effect of 7 min application of ZD7288 30 µM - perforated patch in slice; EC 3 saline plus BL 1 and BL 2 mixes; RT. G: Current-clamp responses to the injection of a hyperpolarizing current step (−60 pA) in a TH-GFP+ cell; black trace recorded in normal [K^+^]_o_ (EC 2), red trace recorded in high [K^+^]_o_ (EC 3);V_rest_ was −64.9 mV and −57.8 mV in normal and high K^+^, respectively; both recordings were performed with BL 1 mix,perforated patch in slice at 26 °C. H: Current-clamp responses to the injection of a hyperpolarizing current step (−56 pA) in a TH-GFP+ cell; all traces were recorded in high [K^+^]_o_ (EC 3) plus BL 1 mix; the, red trace was recorded in the same saline plus 1 mM Cs^+^;V_rest_ was −55.8, −73.2 and −55.3 mV in control, Cs^+^ and washout, respectively;current-clamp recording; perforated patch in slice at 37 °C.

A common attribute of DA neurons in the CNS is their capability to generate rhythmic action potentials even in the absence of synaptic inputs [13]–[15], a feature shared by DA cells in the glomerular layer of the olfactory bulb [16]. In many autorhythmic cells, a key role in the pacemaking process is played by the inward current (I_h_; for a review see [17]), carried by channels encoded by four HCN genes (*h*yperpolarization-activated *c*yclic *n*ucleotide-sensitive *c*ation *n*onselective).In a previous study [16], analyzing the excitability profile of DA PG cells, we failed to detect any significant component activated by hyperpolarization (Fig. 1A), concluding that there were no hyperpolarization-activated currents. On the other hand, an unidentified fraction of rat PG cell showed an evident h-current in normal saline [18]. Our conclusion was that HCN channels were absent in DA PG cells. This conclusion was subsequently strengthened by the observation that, using a comprehensive set of antibodies against all four isoforms, no HCN channels were detected in DA PG cells [19]. It was therefore surprising to observe, later on, an inhibition of spontaneous firing of bulbar DA neuronsby selective blockers of the h-current. We then re-examined the problem, finding that in fact there is an h-current, undetected by our previous investigation due to its small amplitude. This current can be better evidenced with ionic manipulations and, despite its small amplitude, can play a role under physiological conditions. In this paper we describe the properties of this current.

## Materials and Methods

### Animals and surgical procedures

#### Ethic statement

Experimental procedures were carried out so as to minimize animal suffering and the number of mice used. The procedures employed are in accordance with the Directive 86/609/EEC on the protection of animals used for experimental and other scientific purposes, and are approved by the Campus Veterinarian of the Ferrara University.

A total of 102 mice were used. All experiments were performed using the transgenic mice TH-GFP/21–31 line carrying the eGFP gene under the control of the TH promoter [20], [21]. Transgenic mice were identified either by PCR on the genomic DNA extracted from tail biopsies, or -at postnatal day 3 or 4- looking at the fluorescence of the olfactory bulbs transilluminated with a UV source (FBL / Basic-B & N-01; BLS, Hungary; FHS/F-01) and observed with an emission filter (FHS/EF-2G2; BLS, Budapest, Hungary). Transgenic lines were maintained as heterozygous by breeding with C57BL/6J inbred mice.

### Dissociation procedures

Adult mice (30–60 day-old) were used to isolate olfactory bulb neurons. Two solutions were used for the preparation: a dissecting solution and Tyrode's solution. The dissecting medium (DM) contained (in mM): 82 Na_2_SO_4_, 30 K_2_SO_4_, 10 HEPES, 5 MgCl_2_, 10 Glucose, and 0.001% phenol red indicator; pH was adjusted to 7.4 with NaOH and the solution was continuously bubbled with 100% O_2_. Tyrode's solution contained (in mM) 137 NaCl, 5.4 KCl, 1.8 CaCl_2_, 1 MgCl_2_, 5 HEPES, 20 Glucose; the pH was adjusted to 7.4 with NaOH and the solution was continuously bubbled with 100% O_2_. Dissociation of the olfactory bulb by enzymatic digestion and mechanical trituration was performed following the procedure described by Gustincich [22], with minor changes. After dissecting and slicing the bulbs, the small pieces were transferred to a solution containing DM and 3% protease type XXIII (Sigma) for 30–45 min at 37 °C. After enzymatic digestion, the bulbs were transferred to solution containing DM, 1% bovine serum albumin (Sigma) and 1% trypsin inhibitor (Sigma) to stop protease activity (10 min, 37°C). Bulbs were finally suspended in Tyrode's solution and triturated using home-made fire-polished Pasteur pipettes of varying gauges. The cell suspension was centrifuged at 107 g (5 min), and the pellet was resuspended in Tyrode's solution. The dissociated olfactory bulb neurons were plated on glass coverslip previously coated with concanavalin A (1 mg/ml) to allow sedimentation ofthe cells. The cells were maintained at 37°C in an atmosphere of 5% CO_2_/95% air, in DMEM (Dulbecco's modified Eagle medium), supplemented with 10% FBS (fetal bovine serum) and 10%penicillin-Streptomycin. The cells were allowed to set on the glass for at least 12 hour before commencement of recordings.

### Recording conditions

The temperature of the 1-ml recording chamber was controlled using a couple of 39.7 W Peltier devices (RS Components, Milan, Italy) and measured with a high-precision, low mass thermocouple (RS Components).

Current and voltage recordings were acquired with an MultiClamp 700B amplifier (Molecular Devices, Sunnyvale, CA), and a 12 bit A/D–D/A converter (Digidata 1440A; Molecular Devices). Borosilicate glass pipettes (1.5 O.D., 0.87 I.D., with filament; Hilgenberg, Malsfeld, Germany) were pulled with a Zeitz-DMZ puller (Martinsried, Germany) and had a resistance of 4–5 MΩ when filled with standard intracellular (IC) solution; the seal formation was realized with the help of an air pressure controller (MPI, Lorenz Messgerätebau, Katlenburg-Lindau, Germany); the seal resistance was always greater than 2 GΩ. The liquid-junction potential (LJP) of the different solutions was estimated using the junction potential calculator of pClamp (Molecular Devices).

### Solutions

The solutions used had the following composition (mM):

*EC 1 - standard extracellular (EC) saline*: 125 NaCl, 2.5 KCl, 26 NaHCO_3_, 1.25 NaH_2_PO_4_, 2 CaCl_2_, 1 MgCl_2_, and 15 glucose; LJP+3.0 mV.

*EC 2 - modified EC saline (normal K, TEA)*: 105 NaCl, 2.5 KCl, 1.25 NaH2PO4, 20 TEA-Cl, 26 NaHCO3, 1 MgCl2, 2 CaCl2; LJP+3.5 mV.

*EC 3 - modified EC saline (high K, TEA)*: 85 NaCl, 32.5 KCl, 1.25 NaH2PO4, 20 TEA-Cl, 26 NaHCO3, 1 MgCl2, 2 CaCl2; LJP+2.7 mV.

All EC solutions were continuously bubbled with 95% O_2_ and 5% CO_2_; the osmolarity was adjusted at 305 mOsm with glucose.

In the contexts indicated in the text, the following mixes were used:

*BL 1 - synaptic blockers*: recording from slices, the EC solutions always included kinurenic acid (1 mM) and bicuculline (10 µM);

*BL 2 - ion channels blockers*: to isolate the h-current, both in slices and dissociated cells, a mix of blockers (TTX 0.6 µM, Cd2+ 100 µM, andBa2+ 0.5 mM) were normallyadded to the bath, except where indicated.

*standard pipette-filling intracellular (IC) solution*: 120 KCl, 10 NaCl, 2 MgCl_2_, 0.5 CaCl_2_, 5 EGTA, 10 HEPES, 2 Na-ATP, 10 glucose.

The free calcium concentration with this internal solution was calculated to be 16 nM (http://www.stanford.edu/~cpatton/downloads.htm).

For perforated patches, amphotericin B was included in the recording electrode filling solution as perforating agent (200 µg/ml plus 300 µg pluronic F-127). In order to ensure the integrity of the perforated patch, EGTA was omitted from this solution and the concentration of CaCl_2_ was raised to 3 mM in order to monitor possible spontaneous breakups of the perforated patch. Data were collected after the series resistance fell to <50 MΩ.

In all IC solutions the osmolarity was adjusted to 295 mOsm with glucose, and the pH to 7.2 with KOH.

### Data analysis

I_h_ was evoked by a family of hyperpolarizing voltage steps from the holding potential of −40 mV to −130 mV in 10 mV increments. The steps were applied in 10 s intervals. Offline analysis was performed using version 10 of pClamp (Molecular Devices) and version 8 ofOrigin (OriginLab Corporation, Northampton, MA).

When box charts are used to represent data ensembles, the central line indicates the mean, boxes S.E., whiskers min-max values.

### Analysis of current recordings

The I_h_ amplitude was measured as difference between the steady-state currents at the end of test voltage pulses (I_ss_) and the instantaneous currents and the beginning (I_inst_); the latters were measured extrapolating the double exponential fitting the h-currentto the time of the onset of the hyperpolarizing pulse.

Rates of I_h_ activation were determined using the following function (Clampfit 10.2, Molecular Devices): 
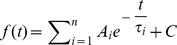
(1)


where *i*  = 1 or 2 (corresponding to single or double exponential fit), *A_i_* is the amplitude of the fitting component(s), *τ_i_* is the time constant(s), and *C* the shift of the fitted trace from zero, i.e. -*A_i_* aligning the baseline to zero.

**Figure 2:**
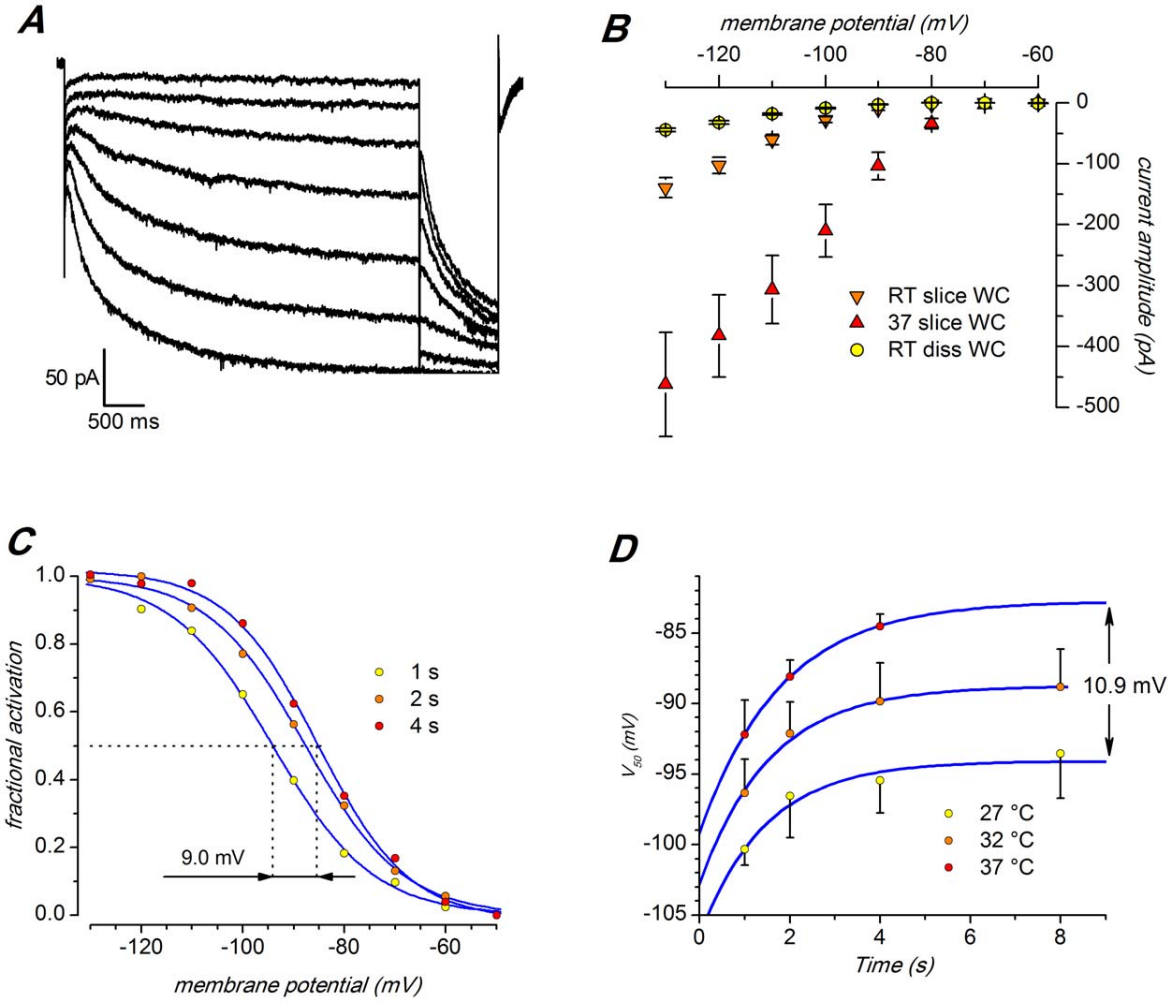
Activation kinetics. A – Representative current traces for the analysis of activation. The membrane was held at −40 mV and then hyperpolarized to test voltages from −60 to −130 mV in 10 mV increments,10 s interval. I_h_ tails were elicited in response to a second pulse to −130 mV, following test voltages (see methods for explanation). EC solution: EC 3, plus BL 1 and BL 2 mixes; 37°C; slice; perforated patch. B: Whole-cell current-voltage relationship of the h-current in different experimental conditions: dissociated cells at RT (○, n = 11); slice, RT(▿, n = 14); slice, 37 °C (▵, n = 7); mean values ± S.E. EC solution was EC 3 plus BL 2 mix in all cases, with the further addition of BL 1 mix in slice preparation. C – Fractional activation of the h-current as in a group of 9 cells as a function of voltage using the protocol shown in A, and with the indicated duration of the hyperpolarizing step (see text for explanation). EC solution: EC 3, plus BL 1 and BL 2 mixes; 37°C; slice; perforated patch. D – Effect of temperature and of the variable duration of the hyperpolarizing step on the midpoint of the h-current. Notice that the change from room temperature (22±1 °C) to 37 °C entails a shift of 10.9 mV of the V_50_. EC solution: EC 3, plus BL 1 and BL 2 mixes; slice; perforated patch.



The activation curve of I_h_ was constructed using a two-step protocol [23]: I_h_ was first activated to a variable degree by a conditioning step, and then fully activated by a second pulse to −130 mV (Figure 2A). The resulting tail current amplitudes were then normalized and fitted by the equation:

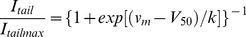
(2)where I_tail_ is the amplitude of the tail recorded at the second pulse, I_tailmax_ is the maximal amplitude of the tails, V_m_ is the membrane potential; V_50_ is the membrane potential for which half of the channels are open (midpoint); *k* is the dependence of the opening of channels by the change of potential (slope).

The temperature coefficients of activation and deactivation time constant are defined as:
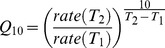
(3)


Thus, for every 10°C of change in temperature there is a *Q*_10_-fold change of the rate analyzed.

Unless otherwise stated, data are presented as means ± s.e.m. Statistical significance of the results was assessed with one-way or two-way analysis of variance (ANOVA), Student's *t* test for paired samples, as indicated. D'Agostino & Pearson omnibus normality test was used; a *P* value of *<*0.05 was considered significant.

## Results

Data are based on recordings from 285 TH+ periglomerular (PG) cells; neurons were selected on the basis of their position around the glomeruli, dendritic arborization within the glomerular neuropil, membrane capacitance (8.54+0.21 pF; n = 285) and input resistance (915.69±31.31 MΩ; n = 248). Dopaminergic PG cells can be differentiated from external tufted cells not only by their large difference in membrane capacitance and input resistance, but also by their different modality of firing, regular in DA-PG cells [16], in bursts in external tufted cells [24]. Short-axon cells have membrane capacitance and input resistance very similar to PG cells, but usually they can be recognized in slice for their position between glomeruli, fusiform shape and dendrites extending to different glomeruli [25].

The transgenic mice used in these experiments,expressing the reporter protein eGFP underthe TH promoter [26], is a well-tested animal model forthe study of dopaminergic neurons[16], [27]–[29] providing a useful tool for examining dopaminergic cellsin the rodent CNS.

A first series of experiments was carried out using perforated patch recordings both in enzymatically dissociated cells and in slices at room temperature. In these conditions, using the standard external saline, hyperpolarizing steps from −40 mV to potentials ranging from -50 to −130 mV (10 mV increments, 10 s interval) caused only small currents, whose amplitudes were of the order of magnitude of purely ohmic components (Fig. 1A). Using an external saline where [K^+^]_o_ was 32.5 instead of 2.5 mM, we observed a measurable current activated by hyperpolarization (Fig. 1B). A fraction of this current could be blocked by Ba^2+^ 500 µM, and was identified as a classical inward rectifier potassium current (K_IR_ type) – this component was not further analyzed in this study. However, a component relatively insensitive to Ba^2+^ persisted (Fig. 1C), which could be suppressed by Cs^+^ 1 mM (Fig. 1D and H; n = 10), a non-specific blocker of the h-current [30], and by two organic compounds known as selective blockers of the h-current, ZD7288 30 µM (n = 5; Fig. 1E–F) [31] and the bradicardic agent S-16257 (ivabradine, 10 µM; n = 4 - not shown) [32], [33].

Rising the external potassium concentration and under current-clamp conditions, the typical sag denoting the presence of an h-current was clearly observable (Fig. 1G), and could be blocked by Cs^+^ 1 mM (Fig. 1H).

Activation in response to hyperpolarizing command potentials, slow kinetics of activation, dependenceon potassium ion concentration and pharmacology, all concur in the unambiguous identification of this current as a typical h-current.

### Kinetics

Hyperpolarizing commands from a holding potential of −40 mV evoked slow inward relaxations (Fig. 2A). The h-current activated slowly and increased magnitude and rate of activation as cells were progressively hyperpolarized, with no sign of inactivation. Two current components were measured during the hyperpolarizing voltage steps: (i) an instantaneous current (I_ins_), obtained at the beginning of the step; (ii) a steady-state current (I_ss_), obtained at the end of the step. The instantaneous current was almost linear along the explored voltage, while the steady-state current increased its magnitude as the membrane potential was made more negative; the h-current amplitude, measured as I_ss_-I_inst_ (see methods) is plotted against voltage in Fig. 2B in different experimental conditions.

The amplitude of the h-current was strongly dependent upon the experimental conditions. First, the current in dissociated cells (○, Fig. 2B) had an amplitude much smaller (30.7%±1.15) than the current measured in slices at the same temperature (▿), and consequently, all the experiments were conducted in slices.

Second, as described in various types of preparation [34]–[36], the kinetics of I_h_ is particularly sensitive to thermic conditions. Figure 2B shows the effect of a temperature increment on the I_h_ amplitude at different potentials: I/V graphs represent the mean current amplitudes in DA cells in slices recorded at room temperature (22±1 °C, ▿) andat 37 °C (▵) as a function of membrane potential. At −130 mV, a 15 °C increase causes a rise in amplitude from −139.02±16.73 pA at 22 °C (n = 14) to −462.51±85.84 pA at 37 °C (n = 7). The average value of Q_10_ for the I_h_ amplitude between −100 and −130 mV is 2.87±0.38. The resulting maximal conductance *g_h_* at 22 and 37 °C is 0.93 and 3.08 nS, respectively.

To determine the I_h_ voltage dependence, activation curves were created fromI_h_tail currents obtained by repolarizing the membrane to a potential at which the h-current was fully activated (−130 mV) after a prepulse at different potentials, as explained in Methods (Eqn. 2). The activation curves were fitted by the Boltzmann function to estimate the potential of half-activation (*V*_50_) and the slope factor (*k*). The point of half activation of the h-current critically depends on the temperature (see below) and on the hyperpolarizing pulse length [37], as measurement errors are more pronounced for slow HCN channels than for fast ones [36], [38]. Therefore, we have analyzed the dependence of the midpoint from the duration of the conditioning command. In nine cells, studied with the double pulse protocol described above, the first command had durations of 1, 2, 4 and 8 s -we also tried the next point in the log scale, 16 s, but the membrane did not tolerate the prolonged hyperpolarizations at the more negative potentials. Increasing the duration of the conditioning pulse induces a significant shift of the steady-state activation curves in depolarizing direction (Fig. 2C): at 37°C, the values of V_50_ is changed from −94.1±2 mV for 1 s stimuli to −84.5±1.22 mV for 4 s without significant changes in the corresponding slopes. The protocol was repeated at 27 and 32 °C, with similar results (Fig. 2D).

**Figure 3:**
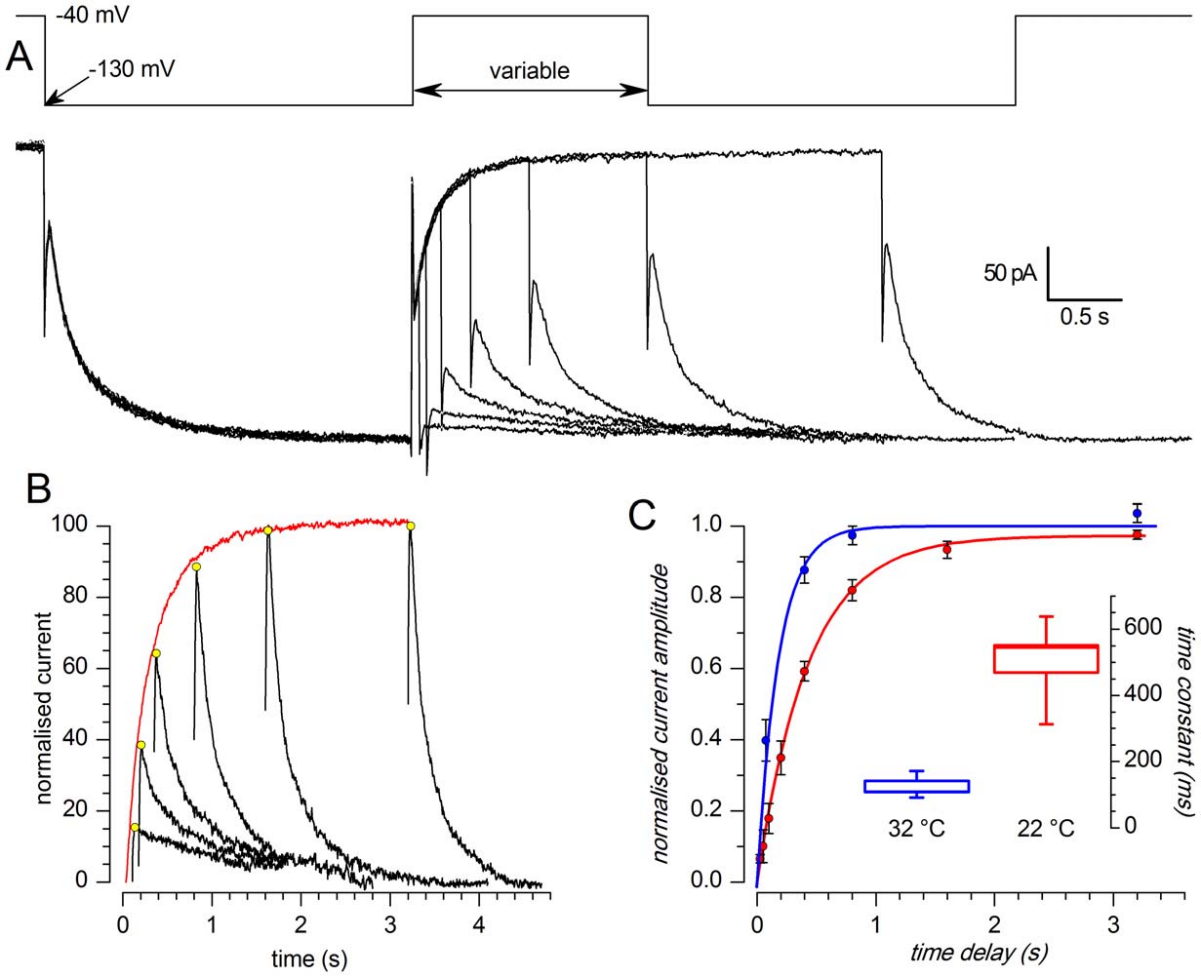
Deactivation kinetics. A: Envelope test during deactivation at −40 mV. After current activation at −130 mV, pulses to −40 mV of variable duration were followed by re-activating steps to −130 mV (see protocol in the top panel). Recording temperature 24 °C. B: The tail at −40 mV was also re-plotted after appropriate scaling (red trace) to better compare its time course with that of the re-activation records envelope shown in panel A. C: Analysis of the deactivation time constant for a group of five cells at 32 (blue) and 22 °C (red); the average time dependence was fitted with the equation I_(t)_ = 1 - exp(-t/τ) (continuous lines); C - inset: box chart of deactivation time constants at 22 and 32 °C; All recordings shown in this figure were made in slice, perforated patches, EC 3 saline plus BL 1 and BL 2 mixes, at the indicated temperatures.



The de-activation time constant was measured using the envelope test [39] shown in Figure 3: from a holding potential of −40 mV, two hyperpolarizing pulses to −130 mV lasting 4 s were imposed, separated by a repolarization to −40 mV of variable length (Figure 3A). In Fig. 3B, I_h_ de-activation at −40 mV and the envelope of re-activation records at −130 mV shown in panel A are displayed together to evidence the similarity of their exponential time course. The values of the tail current amplitudes recorded upon re-activation at −130 mV were normalized, plotted as a function of depolarizing step duration (Figure 3B, C), and the de-activation time constant was calculated by interpolating the experimental points with the exponential function

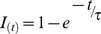



where *I_(t)_* is the normalized current amplitude at time *t*, and τ is the time constant of de-activation at the indicated potential.

#### Effect of temperature

**Figure 4:**
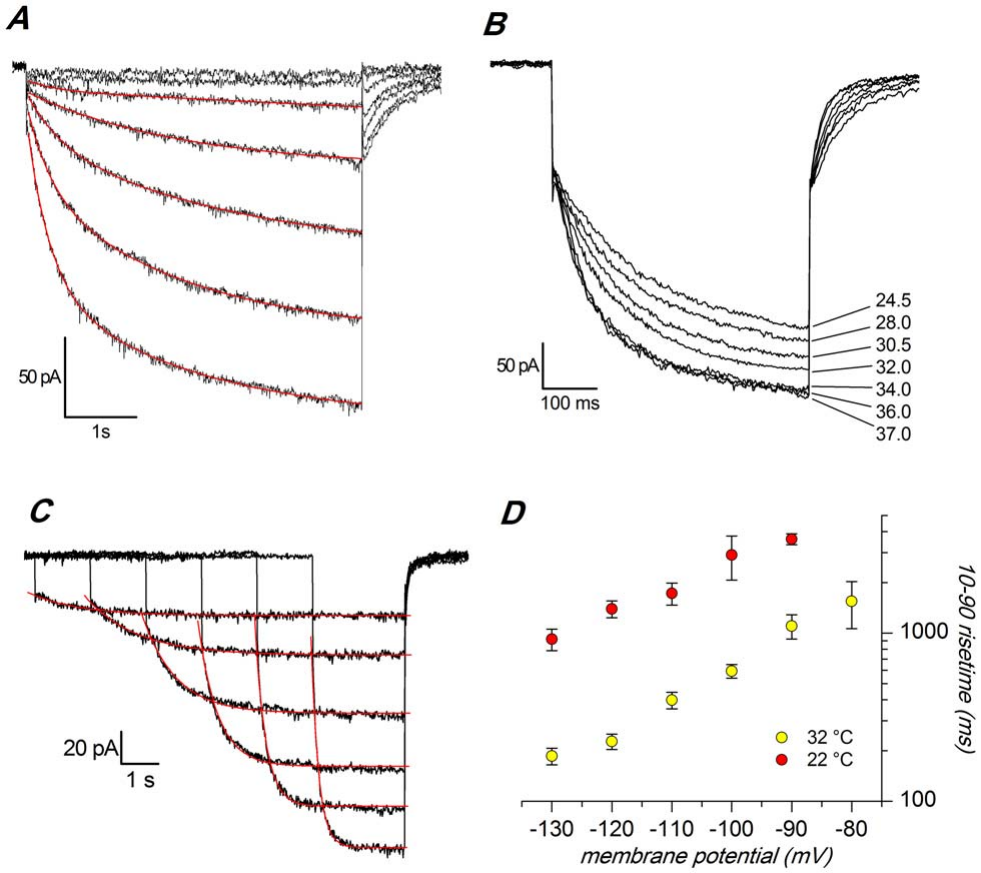
Analysis of time constants. A: Activation at 22 °C: family of current tracings recorded in a single cell in response to hyperpolarizing pulses ranging from −70 to −130 mV from a holding potential of −40 mV; the red line represents the best fit with a double exponential;B: Family of current tracings recorded in a single cell in response to hyperpolarizing pulses from −40 to −130 mV, repeated at the temperatures indicated. C: Activation at 32 °C, using a different protocol minimizing the stress of the membrane at the more negative potentials; D: Comparison of the 10–90 rise time at the two temperatures. All the records shown in this figure were made in slice, perforated patches, EC 3 saline plus BL 1 and BL 2 mixes, at the indicated temperatures.

In various types of preparations, the I_h_ kinetics has been shown to be particularly sensitive to thermic conditions [34]–[36]. The temperature at which electrophysiological recordings are made, affecting both amplitude (Fig. 2B and 4B) and kinetics of I_h_ (Figs. 3C and 4D), is one of the limiting factors in comparing the results; therefore, most of the recordings reported in this study were realized under controlled temperature conditions.

As already reported above, we first observed a temperature-induced significant increase in the h-current amplitude (Fig. 2B and 4B).

We then checked whether the increase of I_h_ at −130 mV could be explained by a shift in the voltage dependency. As seen from the graph (Fig. 2D), the transition from 27 °C (yellow symbols) to 37 °C (red symbols) causes a shift of the steady-state activation curve by about +11 mV: the V_50_, calculated fitting the Boltzmann equation to the experimental points (4s conditioning pulses), is −95.44±2.33 mV at 27 °C (n = 13) and −84.2±1.3 mV at 37 °C (n = 18), (P<0.0001, two-tailed Student *t-*test for unpaired data). No significant changes were observed in the slope of the Boltzmann curve, which was 8.0±0.37 mV at 27 °C and 7.74±0.4 mV at 37 °C.

Temperature does not affect only the total conductance of the h-current (Figure 2B) but also its activation kinetics in two aspects: first the tracings at 22 °C can be accurately fitted only using a double exponential (Figure 4A), whereas at temperatures above 32 °C a single exponential gives an adequate fit (Figure 4C); second, the rate of development of the current, which was increased. As in other preparations, the rise time of the current is strongly affected by temperature, as it can be appreciated at first sight comparing the traces of Fig. 4A and C. However, since at 32 °C there is only one exponential, and at 22 °C two, a comparison of the time courses was possible only comparing the 10–90% rise time. Since the steady state was not always reached due to the instability of the membrane at the more negative potentials, we used the following equations, obtained solving equation 1 for y = 10 and y = 100 after normalization of the total amplitude to 100:

- for a single exponential *t*_90_ = τ ln(^100^/_10_) and *t*_10_ = τ ln(^100^/_90_), where *t*_10_ and *t*_90_ are the times at which the current is developed for the corresponding percentage, and τ is the time constant;

- for a double exponential:

- first the amplitudes of the two exponentials (*A_1_* and *A_2_*) were normalized so that their sum was 100;
- then, Eqn. 1 was solved numerically for *t* using standard numerical methods [40], [41] solving Eqn. 1 for *f(t)* = 90 and 10 (the Matlab code used can be found in the Supplementary material of [42]), thereby obtaining *t*_10_and *t*_90_, respectively.

The comparison of the *t*_10_ - *t*_90_ times at 22 and 32 °C is represented graphically in Fig. 4D, and the corresponding Q_10_, in the range −90 –130 mV, is 4.72, as calculated with Eqn. 3 setting *rate* as (*t*_90_ *–t*_10_).

#### Reversal potential

**Figure 5:**
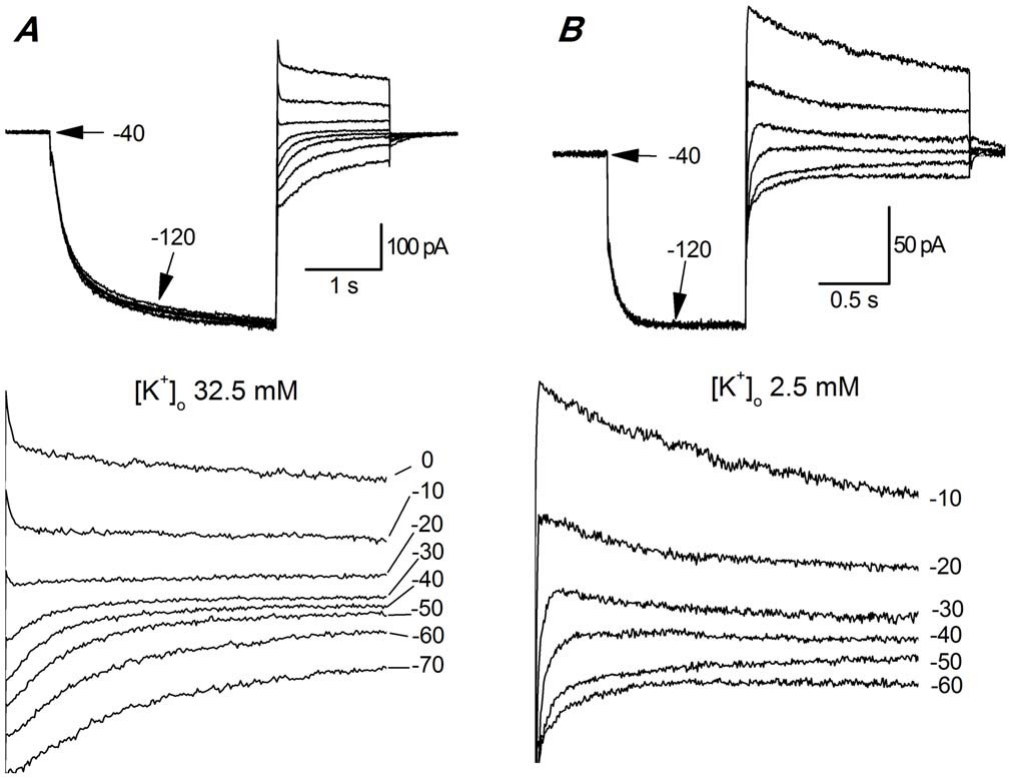
Reversal potential. A, B - measure of I(h) reversal potentials; following activation, I_h_ was deactivated by 1.4 s depolarizing steps to various voltage levels, as indicated in the bottom panels. The expanded decay tails, plotted in the bottom panels, show that the I_h_ reversal potential is around−20 mV in A, and around −40 mV in B. The reversal potentials do not change with temperature (not shown). Recording conditions: *A*: EC 3 plus BL 1 and BL 2 mixes, RT; *B*: EC 2 plus BL 1 and BL 2 mixes, 37 °C; both recordings were made in slice using perforated patches.

Panels A and B of Fig. 5 show current records during protocols used to determine the voltage at which I_h_ reverses. They consist of a series of fixed hyperpolarizing pulses, followed by repolarizations to various levels. The reversal potential did not depend upon the temperature (not shown) but only on sodium and potassium ion concentration. In Figure 5 we show the dependence on K^+^ ions; on average, E_h_ was −20.18±1.67 mV (n = 9) in [K^+^]_o_ 32.5 mM, and -43.95±1.51 mV in standard saline ([K^+^]_o_ 2.5 mM; n = 7); the reversal potential was not temperature-dependent (not shown). From the reversal potential and the h-current amplitude, the maximal conductance could be calculated, giving a value of 1.37 nS in standard saline.

### Pharmacology

#### Blockers

The h-current is sensitive to low concentrations of Cs^+^ (1–2 mM) [30] and to a certain number of organic compounds capable of selectively blocking the h-channels, like ZD7288 [31] and S-16257 (ivabradine) [32], [33]. Cs^+^ 1 mM effectively blocks the h-current (Figure 1D, H); however, as already described for calf Purkinje cells [43], the action of Cs^+^ is clearly voltage-dependent: in the negative region of the I-V curve Cs^+^ induces a channel blockade, whereas at more positive potentials Cs^+^ is ineffective, and sometimes it can even produce the opposite effect, i.e. a current increase (not shown). More selective and completely voltage-independent blockages can be obtained with ivabradine 10 µM (not shown) and ZD7288 30 µM (Figure 1E–F).

#### Role of I_h_ in autorhythmicity

The presence of the h-current, characteristically associated with the pacemaking process in a large number of autorhythmic cell (see [17] for a review) suggests that it could play its archetypal role also in bulbar DA neurons.

**Figure 6:**
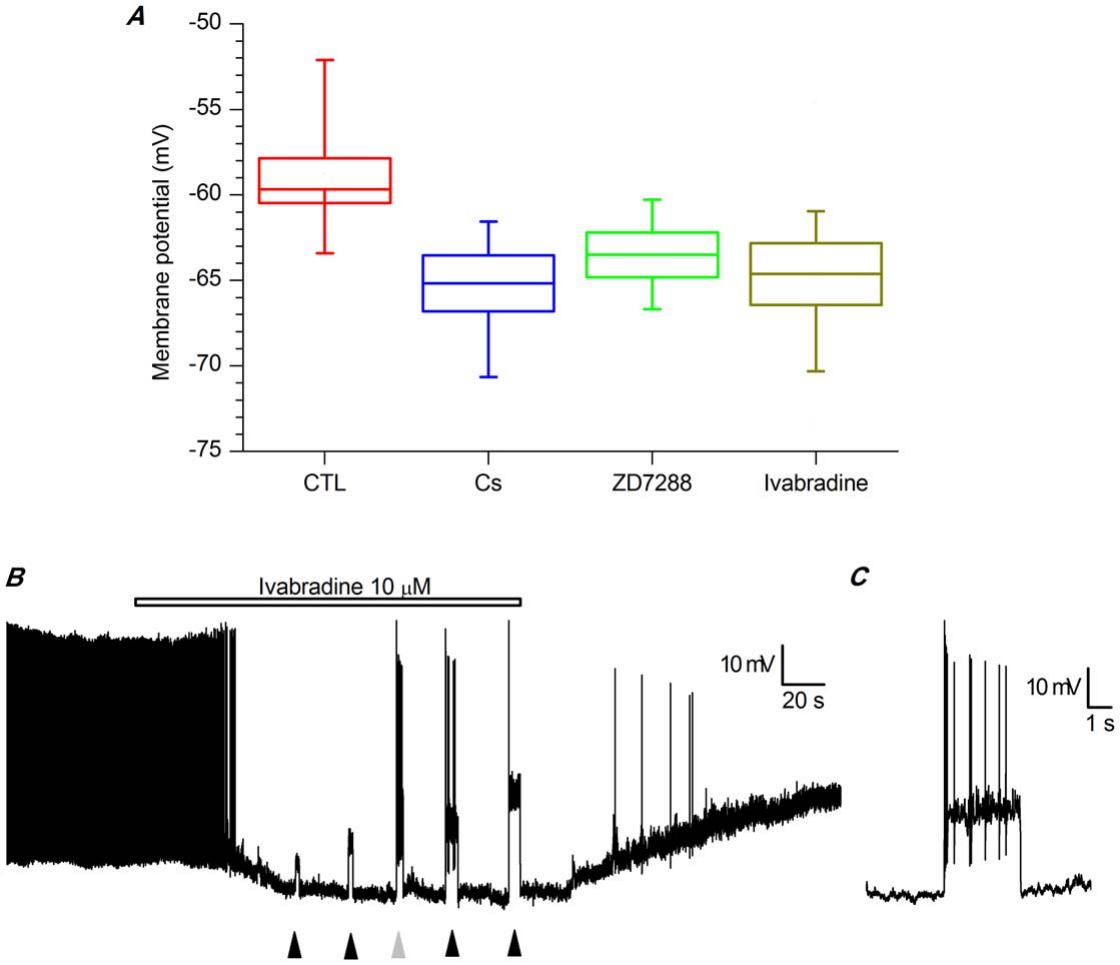
Effect of blockers of h-channels on membrane potential and spontaneous firing. A - Box charts showing the effect of h-channels blockers on resting membrane potential; the recording condition for the experiments represented in this figure were: slice, perforated patch, 37 °C; all differences were significant at the 0.01 level with Student *t*-test paired data analysis. B – Ivabradine (10 µM, bar) block of spontaneous activity. At the times indicated by arrowheads, depolarizing currents of increasing amplitudes (+15, +30, +40, +50, +70 pA, in the order) were delivered. C -Enlargement of the response to the third injection of depolarizing current (grey arrowhead) to show that the block of the h-current does not impair the spontaneous activity, as indicated by the restoration of firing upon forcing the membrane back to resting values. All the experiments shown in this figure were performed using standard saline (EC 1) plus BL 1 mix;slice, perforated patch, 37 °C.

Recording at 37 °C in perforated patches, the block of the h-current by focal application of any drug blocking the h-current (Cs^+^ 1 mM, ZD7288 30 µM or ivabradine 10 µM) induces a hyperpolarization from −58.76±0.9 mV to −65.17±1.64 mV with Cs^+^ (n = 5), −63.5±1.31 mV with ZD7288 (n = 5), and −64.64±1.81 mV with ivabradine (n = 8; Figure 6A), all significant at the 0.01 level with Student *t*-test paired data analysis The effect was rapid and reversible with Cs^+^ and ivabradine (tens of seconds for focal application, 2 min for bath application), slower (about 5 min) and often irreversible with ZD7288.

We then tested whether this blockade represented the evidence of a direct role played by the h-current in the pacemaking mechanism, or just the consequence of the hyperpolarization. In the presence of ivabradine, following the injection of a depolarizing current restoring the resting potential to the value preceding the I_h_ block (grey arrowhead in Figure 6B), spontaneous activity resumed reproducibly and immediately (Figure 6C), proving the absence of any direct involvement of the h-current in autorhythmicity, but also demonstrating that this current has a relevant role in determining the resting membrane potential. The blockage of spontaneous activity for membrane hyperpolarization of a relatively small amplitude (7 mV on average) is not surprising, as cell firing is based on a delicate interplay of conductances that can be easily disrupted by modifications of the resting potential even of small amplitude [16].

These results are different to those observed by Puopolo et al. [44], who, blocking the I_h_ with ZD7288, failed to observe any effect on spontaneous firing and resting potential. We believe that this discrepancy might have several explanations. First, we worked in perforated patch and not in whole-cell configuration; in ruptured patches the h-current is barely discernible, probably because of the known washout of the cytoplasmic compartment, which evidently removes some factors essential for the maintenance of the current. The second explanation might be in the blocker used: we used ivabradine, and Puopolo ZD 7288. The advantage of ivabradine is in its rapidity of action, and in the reversibility of its effect, whereas ZD is slow, and was applied only for a minute. Finally, notice that recordings were done at different temperature (22–24 vs. 34 °C).

#### I(h) modulation by intracellular cAMP

The h current is dually regulated by the hyperpolarization and by cyclic AMP, directly binding to a sequence (cyclic nucleotide binding domain, CNBD) located in the C-terminal segment [45], [46]. We have therefore analyzed the modulatory effect of cAMP on the h-current using a recording configuration (perforated patch with amphotericin B) minimizing the perturbations of the intracellular medium and using a physiological external potassium concentration.

**Figure 7:**
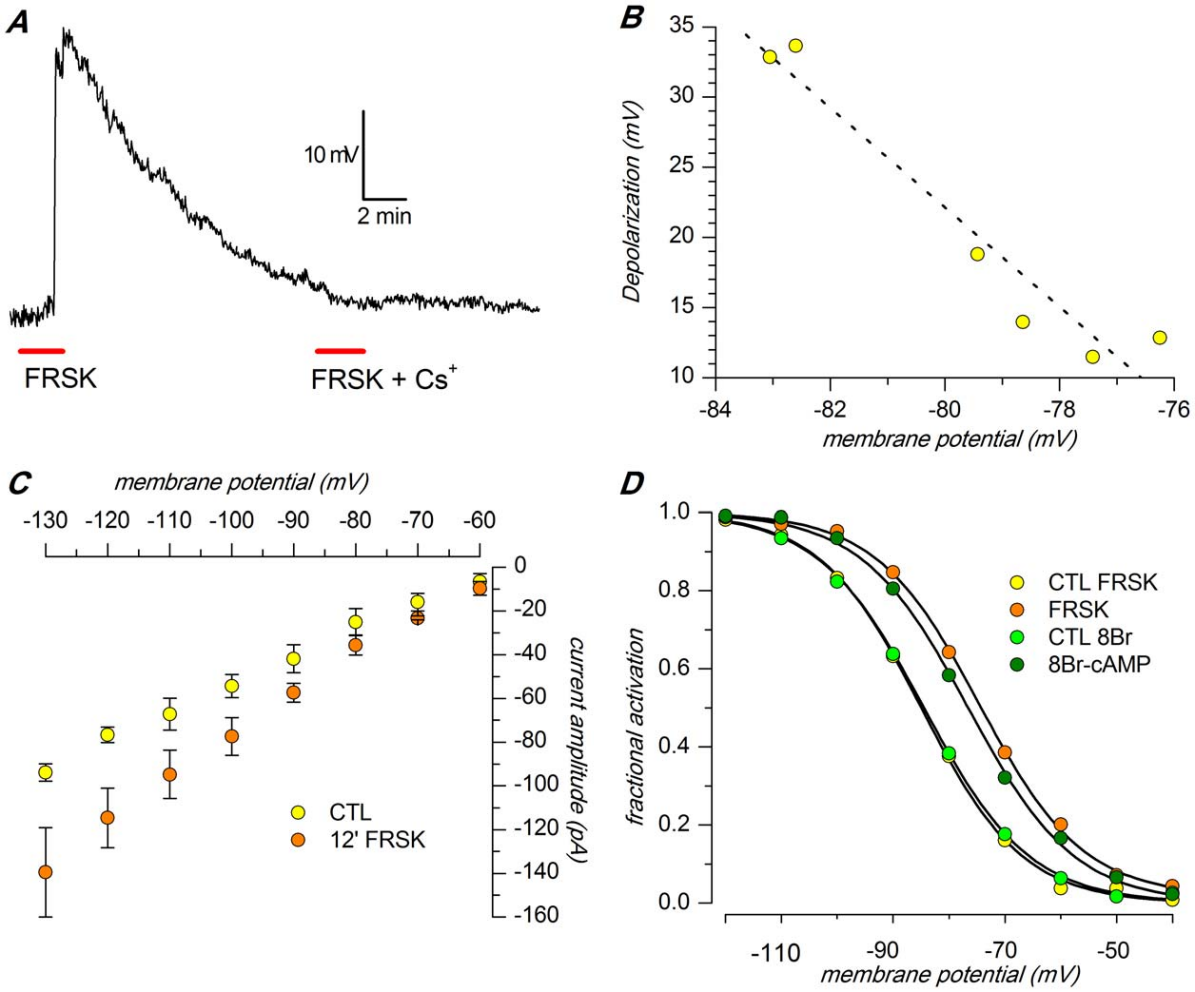
Effect of forskolin. A: Focal application of forskolin (10 µM) and IBMX (0.1 mM) alone and in the presence of 1 mM Cs^+^ at the times indicated by the red bars in current-clamp conditions. Extracellular saline was EC 1 plus BL 1 mix; B:Voltage-dependence of the effect of forskolin on the membrane potential, showing that the depolarization increases at more negative potentials; the cell membrane was manually hyperpolarized before the application of the drug to circumvent spontaneous activity; recording conditions as in A.C: Modification in h-current amplitude after 10' perfusion with forskolin (10 µM) and 8Br-cAMP (10 µM). Extracellular saline was EC 2 plus BL 1 and BL 2 mixes; D: Shift in h-current activation curve following perfusion with forskolin; following treatment with the drugthe midpoint is depolarized of 4.4 mV, and the slope becomes faster (from 7.6 to 5.2 mV); recording conditions as in C. All the experiments illustrated in this figure were performed in slice, perforated patch,37 °C.

Under current clamp conditions and in normal saline, the addition to the extracellular solution of 10 µM forskolin, a classical activator of adenylyl cyclase [47] and0.1 mM IBMX, a phosphodiesterase inhibitor [48], induced a marked depolarization (Fig. 7A); in six cells the average depolarization was 20.53±4.21 mV (n = 6; p<0.005, t-test for paired data), an effect more evident at more negative membrane potentials (Fig. 7B), as expected due to the increasing importance of the h-current at more polarized membrane levels.

Under voltage-clamp conditions and in TEA-normal [K^+^]_o_ saline (EC2), the bath application of 10 µM forskolin and 0.1 mM IBMX, induces a significant increase of I_h_ amplitude (Figure 7C): at −130 mV the current amplitude is −93.8±3.95 pA in control conditions (n = 5), and -139.5±20.4 pA (n = 5) in the presence of increased levels of cAMP;for each tested potential, the increase in current amplitude was statistically significant (p<0.005, ANOVA).

Forskolin promotes a depolarizing shifts of the steady-state activation curve in the depolarizing direction. This could be measured in TEA-normal [K^+^]_o_ saline (EC2), with a variation of V_50_ from −82.44±1.57 mV to −77.11±1.33 (n = 4; significant at 0.005 level; not shown), but it was more evident in high [K^+^]_o_ saline (EC3), a condition favoring more precise measurements of the h-current: in these situations, V_50_ was shifted from −85.26±1.96 to 75.77±2.41 mV (n = 7, p<0.002, ANOVA), and the slope was maintained substantially constant (from 8.77±0.34 to 8.77±0.69, n = 7; Fig. 7D). The experiment was repeated in the same testing conditions but increasing the intracellular cAMP concentration with 10 µM 8Br-cAMP, with results largely superimposable: from −85.36±2.32 to 78.99±3.59 mV (n = 7, p<0.05, ANOVA), and the slope was maintained substantially constant (from 8.85±0.45 to 8.77±0.86, n = 7).

As observed in other systems [42], [49] the increase of intracellular levels of cAMP also changes the activation time course of the h-current onset: in experiments conducted in high [K^+^]_o_ saline (EC3), we observed adecreasing activation time constant both with forskolin (from 141.75±13.81 to 95.58±17.94, n = 7, P<0.02) and with 8Br-cAMP (from 130.27±26.16 to 103.21±23.48, n = 7, P<0.02).

#### I(h) modulation by neurotransmitters

Dopaminergic cells in the olfactory bulb are the target of numerous afferents releasing a variety of neurotransmitters, many of which are known to affect the cAMP pathway, and therefore potentially capable of a modulation of the h-current. Among the others, there are serotoninergic afferents from the ventral and dorsal raphe nuclei [50], noradrenergic input from the locus cœruleus [51], cholinergic inputs from the nucleus of the horizontal limb of the diagonal band [52], and histaminergic inputs from hypothalamus [53]. Furthermore, bulbar dopaminergic cells have been shown to express D2 receptors [54], which could be activated by the dopamine released by the cell itself [28].

**Figure 8:**
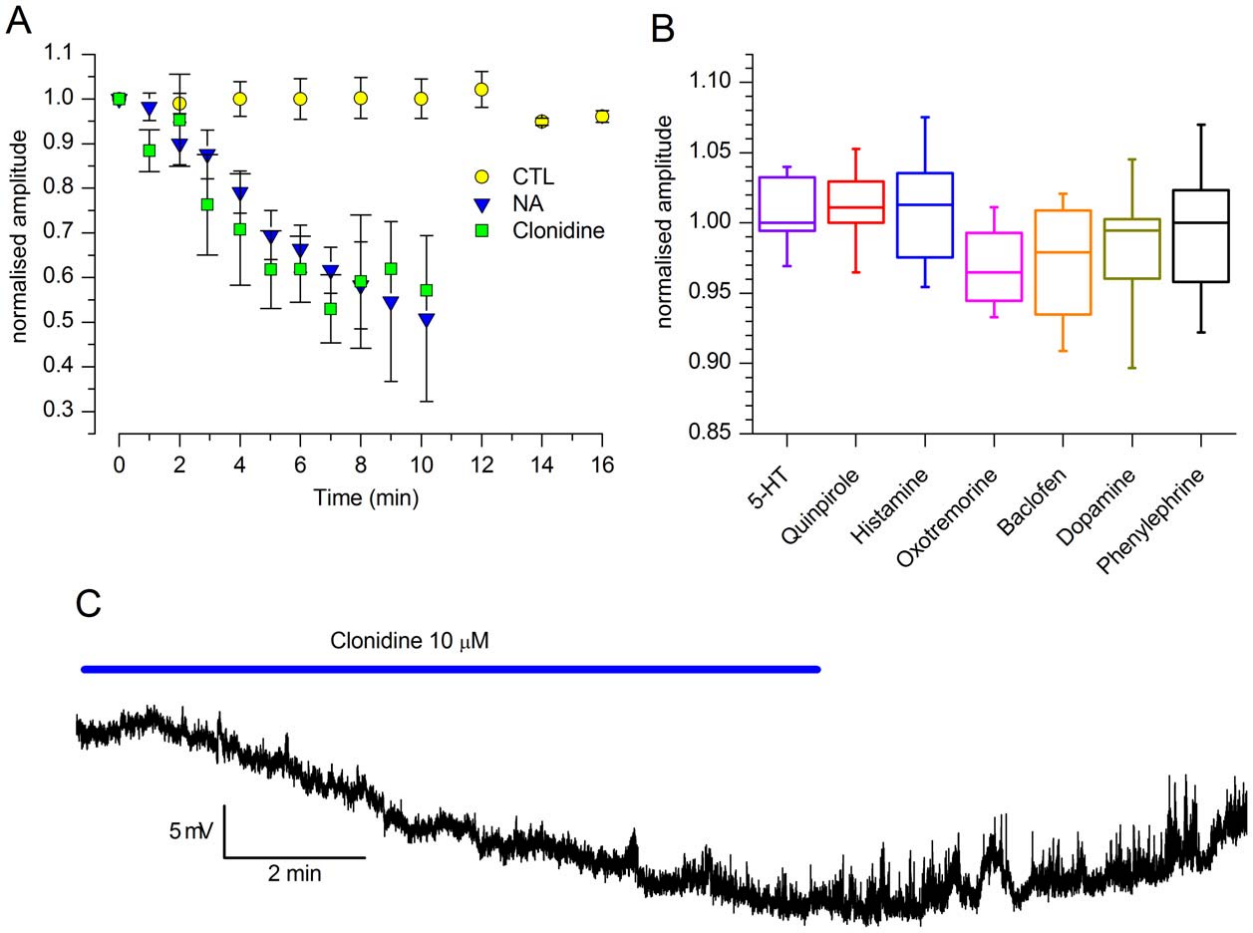
Effect of different neurotransmitters on the h-current. A: effect of NA (100 µM, +1 mM ascorbic acid); the amplitudes at different time points are normalized with respect to the amplitude at time zero; n = 16 (controls) 11 (NA), 6 (clonidine). B: box charts showing the lack of effect of the indicated neurotransmitters;5-HT (50 µM, n = 7),quinpirole (30 µM, n = 14), histamine (10 µM, n = 11), oxotremorine (10 µM, n = 7), baclofen (10 µM, n = 4) and dopamine (100 µM,+1 mM ascorbic acid; n = 8). C: effect of clonidine (α2 agonist, 10 µM) on the resting membrane potential. The membrane was hyperpolarized to −74 mV (by injecting −45 pA) in order to prevent spontaneous firing. All recordings shown in this figure were performed in slice, perforated patch, 37 °C; external saline was EC 3 plus BL 1 and BL 2 mixes for experiments shown in A and B, EC 1 plus BL 1 for the experiment shown in C.

We tested the effects on the h-current amplitude of 5–10 min applications of 5-HT (50 µM), dopamine (100 µM,+1 mM ascorbic acid), quinpirole (D2 agonist, 30 µM), noradrenaline (100 µM,+1 mM ascorbic acid), clonidine (α2 agonist, 10 µM), histamine (10 µM), oxotremorine (muscarinic agonist, 10 µM) and baclofen (GABAb agonist, 10 µM); the results, illustrated in Figure 8, show that only NA affected the h-current, with an inhibition that after 10 min reached 50% of the control level (Fig. 8A), whereas all the other neurotransmitters were ineffective (Fig. 8B). The α2-agonist clonidine reproduced almost exactly the effect of noradrenaline, both in amplitude and time course (Fig. 8A), whereas the α1-agonist phenylephrine was completely ineffective (Fig. 8B). Finally, both NA (not shown) and clonidine induced an evident hyperpolarization of the cell membrane when tested in current-clamp conditions (Fig. 8C).

## Discussion

Two hyperpolarization-activated currents with inward rectifying properties are present in TH-GFP+ neurons. The first has fast kinetics, is permeable primarily to K^+^, is blocked by extracellular Ba^2+^ and Cs^+^, has a voltage-dependence itself dependent on extracellular K^+^ concentration, and has been identified as a classical potassium inward rectifier current (Kir). I_h_ (or I_f_ in cardiac tissue), the second type of hyperpolarization-activated current is a mixed cation current, with a reversal potential substantially positive to E_K_ [55]. I_h_ has a relatively slow activation kinetics, is relatively insensitive to Ba^2+^, and does not show a voltage sensitivity dependent on [K^+^]_o_ [56]. Sensitivity to drugs very selective for I_h_ -like ivabradine or ZD7288-, Ba^2+^ insensitivity, slow kinetics of activation and reversal potential, all suggest that the current described in this paper belongs to the latter class.

A first observation is that the h-current has only a small amplitude in dissociated cells, whereas in slices its amplitude is more than three times larger. A possible explanation is that the h-channels were damaged by the enzymatic treatment, but the possibility of a predominant -albeit not exclusive- location of the channels in the dendritic compartment, largely lost in the dissociation procedure, cannot be excluded. Second, in spite of the prevalent dendritic localization, which is suggestive of a role of the h-channels in the modulation of synaptic input, and despite its small amplitude, the h-current also gives a significant contribution to the resting membrane potential.

### Which population of HCN channels?

Four channel isoforms exist (HCN1-HCN4) that can form homo- or heteromers [17]. Searching in the literature, the expression of TH and of any of the four HCN channels in the olfactory bulb, the situation appears rather confused. The Allen Brain Atlas, based on RT-PCR data, describes only HCN4 channels, whereas others find both HCN2 and HCN4 [57]. Authors using in situ hybridization techniques find either type 1, 2, and 4 [58] or all four of them [59]. The immunohistochemical localization of the different channel subunits has shown that high expression levels of HCN3 can be found in the glomerular layer of the olfactory bulb [60], [61], where HCN3-immunopositive fine dendritic processes and somata are clearly visible;unfortunately the further identification of the cellular subtypes was beyond the scope of those studies.

The search in the literature for co-expression of TH and any of the four HCN channels in the olfactory bulb gives few results. In a recent paper, using acomprehensive set of antibodies against all four isoforms, it was found that all four HCN isoforms are abundantly expressed in the olfactory bulb, where they can be detected in most cell populations, with at least 17 different combinations of staining patterns [19]; however, no HCN channels were detected in TH+ glomerular cells [19], confirming and extending a previous observation limited to the HCN1 subtype [62]. We are notable to explain the reason for the absence of any HCN expression in PG TH+ cells, but certainly, insofar as it is possible to infer from the analysis of the electrophysiological recordings, we expect low levels of expression in these cells, and probably a preferential localization in the dendritic compartment, for which the assignment to a certain cell type or another is not so straightforward.

### Kinetics

At 37 °C, and using 1 s hyperpolarizing pulses, we found a midpoint of activation at -94.1±1.20 mV with a slope of 9.88±0.28 mV (n = 12). In these conditions only 5% of the h-channels would be open at the restingpotential (−65 mV); however, trying different pulse durations, at 37 °C we calculated a h-current steady-state activation midpoint equal to −82.73 mV. The functional implication of these values becomes more evident if one considers that, assuming a slope of 9.2 (the slope shows only a modest increase with change in conditioning step), at −65 mV about 12.7% h-channels are open. The fraction is small but, since the input resistance of these cells is high (915.69 MΩ, personal observation), is sufficient to provide a significant depolarizing contribution to the resting membrane potential. Accordingly, pharmacological block of the h-current with Cs^+^, ivabradine or ZD7288, induces a mean hyperpolarization of 7 mV (Fig. 6A), thus stopping the spontaneous firing. A severe restriction of spontaneous activity following a membrane hyperpolarization of this entity is not surprising, as the cell firing is based on a delicate interplay of conductancesthat can be easily disrupted by changes of a few millivolts of the resting membrane potential [16].

### Pharmacology

#### I(h) dependence on cAMP

The h-channels are directly activated by cyclic nucleotides [45], and -accordingly- we observed a 48.7% increase of I_h_ amplitude in presence of 10 µM forskolin and 0.1 mM IBMX (Fig. 7C). This effect was at least in part sustained by a positive shift of the steady-state activation curve, whose midpoint was moved in the depolarizing directionby 5.33 mV.

The increase of intracellular cAMP induced a depolarization of the membrane which was positively correlated with the resting membrane potential: the larger the membrane polarization, the larger the depolarization induced by the intracellular increase of cAMP Fig. 7B), an effect which can be easily explained by the higher degree of activation (and hence by the greater importance) of the h-current at more negative potentials.

#### I(h) dependence on neurotransmitters

Dopaminergic cells in the olfactory bulb receive numerous afferents releasing a large variety of neurotransmitters, many of which are known to affect the cAMP pathway, and therefore potentially capable of a h-current modulation. We tested several of them, including monoamine (dopamine, serotonin, histamine), and metabotropic cholinergic and GABAergic agonists (oxotremorine and baclofen). All these neurotransmitters do have some effect on the excitability profile of bulbar dopaminergic cells, but none of them showed any effect on the amplitude of the h-current.

The only neurotransmitter showing an effect on the h-current amplitude is NA, which causes inhibition; the effect, that could be replicated by an α2 agonist, is similar to what has been described in L4 and L5 rat dorsal root ganglion neurons [63]. Also in midbrain DA neurons NA inhibits the h-current, either with cAMP-independent mechanisms (activation of the PKC pathway, as in VTA [64]) or due to space-clamp effect (as in *substantia nigra* [65]). There are, however, also reports of enhancement of I_h_ by NA, as in thalamic neurons [66] and in CA1 hippocampal *stratum oriens-alveus* interneurons [67], but without a clear indication of the underlying mechanism.

The OB receives a dense projection from the pontine nucleus locus cœruleus (LC), the largest collection of NA-containing cells in the brain, from where an estimated 40% of neurons project to the OB [68], and which is the exclusive source of NA innervation of the OB [51], [69]. NA fibers preferentially target the internal plexiform layer and the granule cell layer, and, to a lesser extent, the mitral cell layer and the external plexiform layer [51]. The functional role of NA in adult rodent olfactory bulb has been accurately reviewed recently [70]: converging data from electrophysiological studies of cellular-membrane properties of OB neurons and behavioral assays of perception indicate that NA affects the olfactory bulb network improving odor detection and discrimination. Most of the available electrophysiological data concerning the NA effect in different cell types of the olfactory bulb are limited to the granule and mitral cell layer. As for the glomerular layer, despite the fact that all NA receptor types have been localized in this region [71]–[74], no data concerning possible modulation roles of NA inputs are available. Our observation, therefore, would be the first reporting an NA action in a specific subpopulation of glomerular cells, and is well in line with the general effect of NA: dopamine released by glomerular dopaminergic cells is known to inhibit glutamate release from olfactory nerve terminals [11]. Therefore, an inhibition of the h-current, with its hyperpolarizing effect, would decrease the DA-mediated tonic inhibition of olfactory nerve terminals, in line with the general positive influenceof NA on odor detection and discrimination.
